# TMEM207 hinders the tumour suppressor function of WWOX in oral squamous cell carcinoma

**DOI:** 10.1111/jcmm.13456

**Published:** 2017-11-22

**Authors:** Katsuaki Bunai, Hiroshi Okubo, Kimika Hano, Keisuke Inoue, Yusuke Kito, Chiemi Saigo, Toshiyuki Shibata, Tamotsu Takeuchi

**Affiliations:** ^1^ Department of Oral and Maxillofacial Surgery Gifu University Graduate School of Medicine Yanagido Gifu Japan; ^2^ Department of Pathology and Translational Research Gifu University Graduate School of Medicine Yanagido Gifu Japan

**Keywords:** WWOX, TMEM207, HIF‐1α, aerobic glycosylation, oral squamous cell carcinoma, Warburg effect

## Abstract

The WW domain‐containing oxidoreductase (WWOX) functions as a tumour suppressor in oral carcinogenesis. As aberrant TMEM207 expression may lead to tumour progression by hampering the tumour suppressor function of WWOX in various cancers, we explored the expression and pathobiological properties of TMEM207, focusing on the WWOX‐mediated regulation of the HIF‐1α pathway in oral squamous cell carcinoma (OSCC). TMEM207 immunoreactivity was detected in 40 of 90 OSCC samples but not in neighbouring non‐tumorous epithelial tissues. Moreover, TMEM207 expression was significantly correlated with lymph node metastasis and poor prognosis. An *in situ* proximal ligation assay demonstrated the colocalization of TMEM207 and WWOX in invasive OSCC cells, especially glycogen‐rich ones. Enforced expression of TMEM207 abrogated the binding of WWOX to HIF‐1α, increased HIF‐1α and GLUT‐1 expression, even under normoxic conditions, and promoted tumour growth in a xenoplant assay using SAS tongue squamous cancer cells. In contrast, TMEM207 knockdown decreased GLUT‐1 expression in two OSCC cell lines. As a whole, our findings indicate that the aberrant expression of TMEM207 contributes to tumour progression in OSCC, possibly *via* promoting aerobic glycolysis.

## Introduction

The incidence of OSCC increased in recent years 1. Despite recent advances in chemotherapy and radiotherapy, the prognosis of advanced OSCC is still poor 2. To develop new targeted molecular therapies, it is important to unravel the molecular pathways of oral mucosa carcinogenesis 3.

Cancer cells are known to consume more glucose and produce more lactic acid than normal cells, even in normoxic conditions 4. This phenomenon is called the Warburg effect and is caused by aerobic glycosylation 5. OSCC also presents an aerobic glycosylation phenotype, resulting in poor prognosis 6. The hypoxia‐inducible transcription factor 1α (HIF‐1α) and the glucose transporter 1 (GLUT‐1) are key contributors to the Warburg phenotype 7 and are closely related to the nodal metastasis of OSCC 8, 9.

The WWOX exerts tumour suppressor activity in many cancers 10. The loss of WWOX function is believed to drive carcinogenesis in the oral squamous epithelium, probably by augmenting aerobic glycolysis in malignant tumour cells, as WWOX participates in the degradation of the HIF‐1α protein under normoxic conditions 11, 12, 13.

TMEM207 plays a role in the progression of gastric signet‐ring cell carcinoma by binding to WWOX and abrogating its tumour suppressor function 14. Exogenous TMEM207 expression in cutaneous hair follicle bulge cells results in the development of a cutaneous appendage tumour 15. TMEM207 expression is also related to tumour progression in colon mucinous carcinoma and clear renal cell carcinoma 16, 17. Moreover, TMEM207 appears to positively correlate with renal cystogenesis 18. These reports suggest that TMEM207, together with other proteins that transmembrane proteins 19, 20, 21, 22, may comprise a tumour‐associated family of transmembrane proteins. Here, we aimed to unravel the expression of TMEM207 and subsequently to examine the pathobiological property of TMEM207 to ask whether TMEM207 could be a candidate for molecular targeting.

In this study, we evaluated the TMEM207 expression in OSCC tissue specimens by immunohistochemical staining and found that TMEM207 expression was significantly related to worse prognosis of the patients with OSCC. A subsequent *in situ* proximal ligation assay demonstrated that WWOX and TMEM207 were colocalized in the cytoplasm of OSCC cells, especially those cells with a clear, glycogen‐rich cytoplasm. Co‐immunoprecipitation assays suggested that the binding of TMEM207 to WWOX inhibited the interaction between WWOX and HIF‐1α, thereby hampering the degradation of HIF‐1α under normoxic conditions. siRNA‐mediated silencing of TMEM207 impaired GLUT‐1 expression in cultured OSCC cells. Moreover, enforced expression of TMEM207 increased tumour progression in a xenoplant assay.

These findings suggest a novel relationship between TMEM207 and WWOX‐mediated aerobic metabolism in OSCC.

## Materials and methods

### Ethics statement

This study was conducted in accordance with the ethical standards of the Helsinki Declaration in 1975 and approved by the Institutional Review Board of the Gifu University Graduate School of Medicine (App. # 28‐524). Archival paraffin‐embedded tissues that had been surgically resected from patients were used in this retrospective study. The need for written informed consent was waived by the Institutional Review Board. However, according to the advice of the Board, patients or guardians were contacted and given the option to refuse the use of their tissue specimens.

### Antibodies

Rabbit anti‐WWOX and anti‐GAPDH antibodies were obtained from Sigma‐Aldrich (St. Louis, MO, USA), while the rabbit anti‐HIF‐1α and anti‐GLUT‐1 were purchased from GeneTex (Irvine, CA, USA) and Spring Bioscience (Pleasanton, CA, USA), respectively. In this study, we mainly used a monoclonal antibody recognizing the synthetic peptide VNYNDQHPNGW (a.a. 40–50 of TMEM207), whereas an affinity‐purified rabbit antibody against human TMEM207 was applied for confirmation. The detailed procedure for the preparation and characterization of the two anti‐TMEM207 antibodies was described previously 14, 15.

### Immunohistochemical staining

We excluded the tissue specimens which were treated by decalcification in this study. All 90 invasive OSCC tissue specimens were surgically obtained, fixed in 10% buffered formalin and embedded in paraffin. Staining was performed as described previously 23. Briefly, antigen retrieval of deparaffinized sections was performed by autoclaving for 15 min. with 10 mM citrate (pH 6.0) for TMEM207 and 0.25% trypsin (10 min. at 37°C) for WWOX. Tissues were incubated in 10% normal horse serum for 30 min. at room temperature (RT) and subsequently with anti‐TMEM207 overnight at 4°C or anti‐WWOX for 1 hr at RT. In the case of the anti‐GLUT‐1 antibody, tissues were incubated for 30 min. at RT. We employed the ImmPRESS Polymerized Reporter Enzyme Staining System (Vector Laboratories Inc., Burlingame, CA, USA). In all cases, samples were considered positive when more than 10% tumour cells exhibited staining after examining five high‐power fields in one tissue section. No signal was produced when tissues were incubated with TMEM207 pre‐bound with the immunizing peptide, confirming the specificity of the method.

Comparisons of the TMEM207 expression and clinical pathological data were examined for statistical significance using the Fisher's exact test.

### Survival curves

Survival curves were drawn using the Kaplan–Meier method, and the differences in survival rates were compared using the log‐rank test for univariate survival analysis.

### Proximal ligation assay

The detailed procedure for performing the proximal ligation assay using the Duolink *In Situ* Detection Reagents Brightfield Kit (Sigma‐Aldrich) was previously described 16. After autoclaving for 15 min. with 10 mM citrate (pH 6.0), tissue slices were incubated first in blocking buffer for 30 min. at RT and then with 1 μg/ml rabbit anti‐WWOX and mouse anti‐TMEM207 antibodies overnight at 4°C. Subsequently, slides were treated with secondary antibodies conjugated with unique DNA fragments. After ligation and rolling circle amplification, the interaction signals were developed with horseradish peroxidase and NovaRED horse radish peroxidase substrates (Vector Laboratories Inc,) according to the manufacturer's protocol. As a negative control, some slides were incubated with antibodies pre‐bound with the corresponding antigens (the immunizing peptide for anti‐TMEM207). No signal was observed, confirming the specificity of the method.

### Cells, plasmid, transfection and siRNA‐mediated RNA interference

Three human OSCC cell lines were used, namely SAS (obtained from the RIKEN cell bank), SCC‐9 and CHU‐2 (maintained in our laboratory). Detailed procedures, including the preparation of the expression vector for TMEM207, were described previously 14. Briefly, the full coding sequence of the human *TMEM207* gene was amplified by PCR from kidney cDNAs (TaKaRa, Otsu, Japan), subcloned into the pTarget vector (Promega, Madison, WI, USA) and verified by sequencing on the ABI 310 Autosequencer (Perkin‐Elmer, Waltham, MA, USA). The resulting vector designated the ‘TMEM207 expression vector’.

SAS cells were transfected using *N*‐[1‐(2,3‐dioleoyloxy)propyl]‐*N,N,N*‐trimethylammonium methylsulphate (DOTAP) transfection reagents (Boehringer Mannheim, Indianapolis, IN, USA) as previously described 14. Briefly, cells were seeded at approximately 50% confluency in 10‐cm culture dishes. The next day, the culture medium was replaced with 5 ml of Opti‐MEM (Invitrogen, Carlsbad, CA, USA), and then 5 mg of BglII‐linearized TMEM207 expression vector was added. Colonies resistant to 600 μg/ml G418 (Gibco BRL, San Francisco, CA, USA) were selected after 3–4 weeks and subcultured as described previously 14. Three independent clones of TMEM207‐expressing SAS cells were established and confirmed by Western blotting. Cells transfected with the empty vector were used as negative controls.

TMEM207 knockdown was performed as described previously 14. Three siRNAs (SI04341981, SI04286849, SI04277770 and SI04208344; Qiagen, Valencia, CA, USA) or a GFP‐siRNA duplex non‐silencing control (target sequence: 5′‐CGGCAAGCUGACCCUGAAGUUCAU‐3′) were transfected into SCC‐9 or CHU‐2 cells using Lipofectamine RNAiMAX (Invitrogen) in accordance with the manufacturer's instructions. Forty‐eight hours post‐transfection, the cells were harvested and used for subsequent studies. A significant fall in *TMEM207* mRNA levels indicating successful knockdown was achieved with two of the three tested siRNAs, namely SI04341981 (target sequence AACACCCTAATGGCTGGTATA) and SI04277770 (target sequence CACTAGTATCCAAACAGGCAA).

### Reverse transcription, PCR and quantitative real‐time reverse transcription PCR

Synthesis of cDNA from total RNA and subsequent PCR was performed with a Reverse Transcription Polymerase Chain Reaction (RT‐PCR) Kit (TaKaRa) as previously described 14. Real‐time PCR was performed with a SYBR Green Reaction Kit according to the manufacturer's instructions (Roche Diagnostics, GmbH, Mannheim, Germany) on a LightCycler (Roche Diagnostics).

The following primers were used for real‐time RT‐PCR: *TMEM207*, PPH12073A‐200 (Qiagen); *GLUT‐1* forward 5′‐TGCTTGTGGATTGAGGGTAGGA‐3′; *GLUT‐1* reverse 5′‐AAGTCTAAGCCGTTGCAGTGG‐3′; *GAPDH* forward 5′‐GAAATCCCATCACCATCTTCCAGG‐3′; *GAPDH* reverse 5′‐GAGCCCCAGCCTTCTCCATG‐3′. The samples were cultured in triplicate, and the expression of each target gene was analysed using the 2^(−ΔΔCT)^ method 24. The ΔCT values were normalized to *GAPDH* for each triplicate set in both the negative control (the siRNA‐treated group) and the three si*TMEM207*‐treated groups. Values for each of the three target genes were expressed as fold change relative to the ones of the control group (set to 1.0). Standard deviations were computed for the triplicate sets. In addition, Student's *t*‐tests were performed to determine significant differences among groups with *P *<* *0.05 considered statistically significant.

### Western immunoblotting and co‐immunoprecipitation (co‐IP) assays

This procedure was performed as modified by Towbin *et al*. 25. After SDS‐PAGE (sodium dodecyl sulphate polyacrylamide gel electrophoresis), proteins were transferred to polyvinylidene difluoride membranes (Millipore Co., Bedford, MA, USA), blotted with various antibodies and visualized using the Western Blotting Detection Kit (Promega).

Prior to co‐IP, cells were treated with MG‐132 (1 mM) for 4 hrs to stabilize HIF‐1α. The soluble fraction of their lysates was isolated by centrifugation, incubated with a rabbit anti‐WWOX antibody for 2 hrs at 4°C, mixed with protein G‐Sepharose beads (Sigma‐Aldrich) and incubated for 30 min. at 4°C. The beads were washed 4× with the CelLytic M Cell Lysis Reagent (Sigma‐Aldrich) containing a proteinase inhibitor cocktail (Nacalai, Kyoto, Japan). Immunocomplexes were eluted by boiling the samples for 3 min. in SDS sample buffer containing 10 mM dithiothreitol and subjected to SDS‐PAGE followed by Western immunoblotting with a rabbit anti‐HIF‐1α antibody. Finally, the blot was incubated with alkaline phosphatase conjugated with anti‐rabbit IgG (Fc) (Promega).

### Cell proliferation assay and xenografts of SAS cells

Cell proliferation was evaluated as previously described 15. Briefly, 1 × 10^4^ cells were cultured on tissue culture dishes in triplicate. After 24, 48 and 72 hrs, live cells were counted. The assay was repeated twice. Statistical analysis was performed with unpaired Student's *t*‐tests. *P *<* *0.05 was considered significant.

TMEM207‐expressing or control SAS tongue squamous cell carcinoma cells (3 × 10^6^) were subcutaneously injected into the dorsal flank of 8‐week‐old BALB/c nude mice (Charles River Laboratories, Shizuoka, Japan). The length and width of tumours were measured using calipers with a precision of 0.5 mm. The tumour volume was calculated using the following formula: volume=(d1×d2×d3)×0.5236 , where d1, d2 and d3 represent the three orthogonal diameter measurements. Twenty days after inoculation, the xenografts were excised and subjected to formalin‐fixation and paraffin‐embedded tissue sectioning for histopathological analysis. The experimental protocol was approved by the Animal Care Committee of the Gifu Graduate School of Medicine. Statistical analysis was performed with unpaired Student's *t*‐tests. Values of *P *<* *0.01 were considered significant.

## Results

### Expression of TMEM207 in OSCC was related to poor prognosis of patients

We first examined TMEM207 expression in archival pathological tissue specimens of OSCC. As summarized in Table 1, the Fisher's exact test indicated that the overall survival rate of patients with TMEM207 immunoreactivity was significantly lower than that of TMEM207‐negative groups. TMEM207 expression was also related to lymph node metastasis but not to tumour size, age or gender.

**Table 1:** Summary of the clinicopathological characteristics of TMEM207 expression in OSCC

		TMEM207		*P* value
		Positive	Negative	
Age				
<60	63	29	34	0.817
≧60	27	11	16	
Age range: 32–90; Median: 70; Mean ± S.D.: 65.93 ± 14.67 (years)				
Gender				
Male	46	23	23	0.297
Female	44	17	27	
T classification				
T1/T2	71	32	39	1
T3/T4	19	8	11	
Lymph node metastasis				
N0	50	16	34	0.011
N1N2	40	24	16	
Vital status				
Survival	63	21	41	0.003
Death	27	19	9	

**Figure 1:**
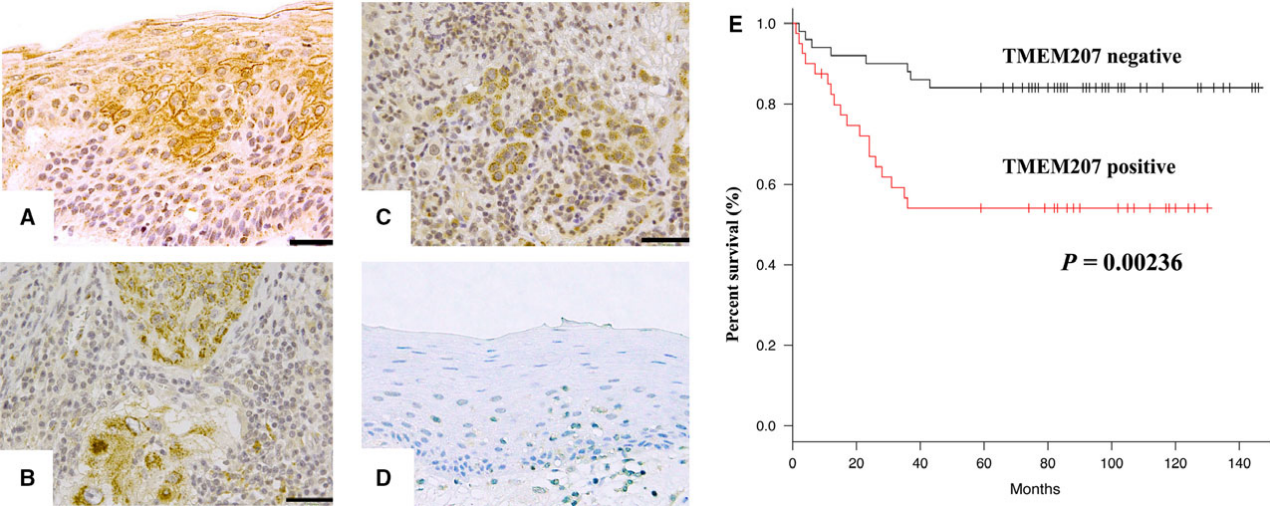
TMEM207 expression in invasive oral squamous cell carcinoma (OSCC) tissue specimens is positively correlated with the overall survival rate. (**A**) TMEM207 immunoreactivity was observed in the membranes of dysplastic epithelial cells neighbouring invasive squamous cell carcinoma cells. Scale bar: 50 μm. (**B**–**C**) Immunoreactivity was detected in the cytoplasm of invasive squamous cell carcinoma cells. Scale bar: 50 μm. (**D**) Note the little to no TMEM207 immunoreactivity in non‐tumorous oral mucosal epithelium. (**E**) Survival curves were drawn using the Kaplan–Meier method. The overall survival of patients with TMEM207 immunoreactivity was statistically worse than that of TMEM207‐negative patients. (*P *=* *0.00236, log‐rank test).

Representative immunohistochemical staining results are shown in Figure 1. TMEM207 immunoreactivity was observed in 40 of 90 invasive squamous cell carcinoma tissue specimens. Notably, we observed TMEM207 immunoreactivity at the cell surface membranes of the dysplastic epithelium neighbouring the OSCC cells (Fig. 1A), while the signal was cytoplasmic in the carcinoma cells themselves (Fig. 1B and C). We did not observe significant TMEM207 immunoreactivity in the non‐tumorous stratified squamous epithelium of the oral mucosa (Fig. 1D). Use of the affinity‐purified rabbit antibody against human TMEM207 instead of the monoclonal anti‐TMEM207 antibody gave similar results.

Survival curves drawn using the Kaplan–Meier method also indicated that the overall survival rate of patients with TMEM207 immunoreactivity was significantly worse than that of TMEM207‐negative groups (*P *=* *0.00236) (Fig. 1E).

### Colocalization of TMEM207 and WWOX in invasive squamous cell carcinoma

Subsequently, we examined the relation between TMEM207 and WWOX, especially focusing on WWOX‐mediated aerobic glycosylation. To determine this relationship, we further examined the OSCC tissue specimens with a clear, glycogen‐rich cytoplasm by immunohistochemistry.

**Figure 2:**
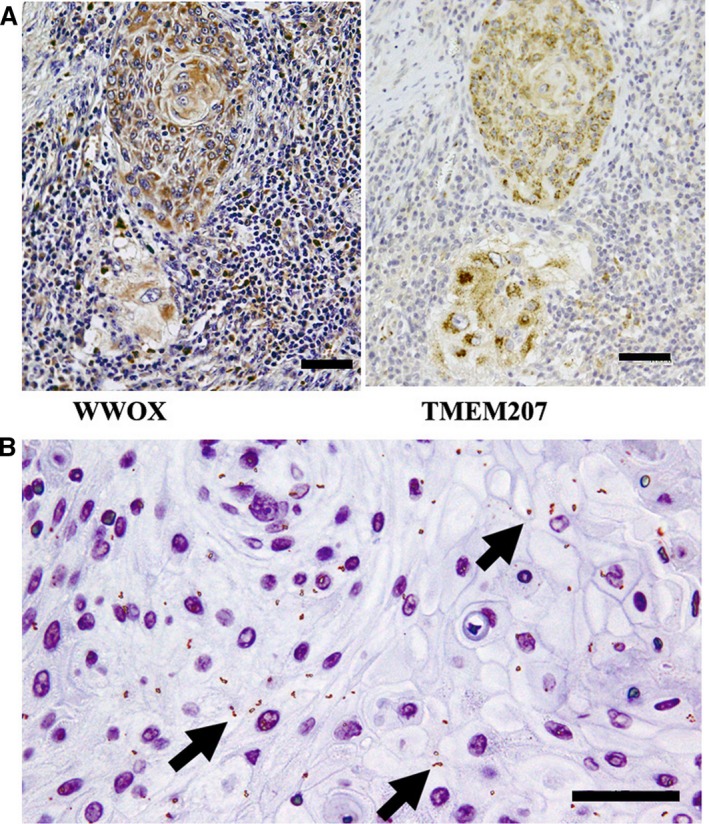
WWOX and TMEM207 are colocalized in invasive squamous cell carcinoma cells (**A**) Representative case of OSCC sample in which both WWOX and TMEM207 were expressed in the invasive squamous cell carcinoma cells. Both WWOX and TMEM207 immunoreactivity were cytoplasmic in these cells. (**B**) Fine granular positive signals (indicated by arrows), which represented overlapping signals for a rabbit anti‐WWOX antibody and murine monoclonal anti‐TMEM207 antibody, as displayed in the *in situ* proximal ligation assay. Scale bar: 50 μm.

WWOX immunoreactivity was found in 22 of 30 OSCC tissue specimens. Notably, 12 of 22 WWOX‐expressing OSCC cells also showed TMEM207 immunoreactivity. The signal was localized in the cytoplasm of the cancer cells (Fig. 2A), as had been the case with anti‐TMEM207.

Subsequently, an *in situ* proximal ligation assay was performed to confirm that WWOX and TMEM207 were colocalized in the cytoplasm of OSCC cells. A strong signal was observed (Fig. 2B), indicating close localization (within approximately 40 nm) or the binding of WWOX and TMEM207. Notably, the signal was most abundant in OSCC cells with a clear, glycogen‐rich cytoplasm. Dysplastic epithelial cells only displayed a weak signal.

### TMEM207 expression decreased the binding of WWOX to HIF‐1α

**Figure 3:**
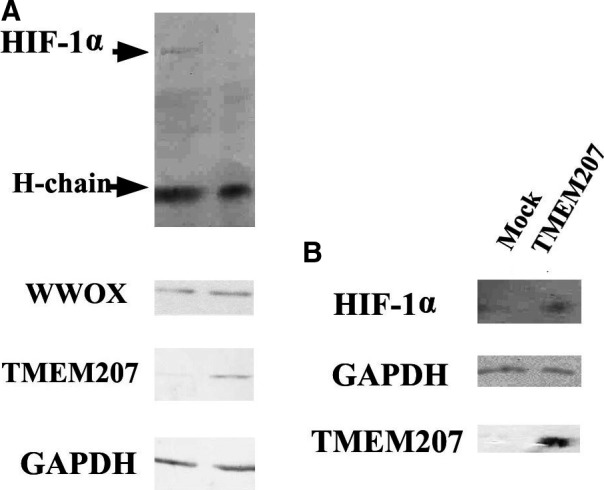
Enforced TMEM207 expression abrogates the binding of WWOX to HIF‐1α (**A**) Representative result of immunoprecipitation of TMEM207‐expressing and control SAS cells with anti‐WWOX, followed by Western immunoblotting with anti‐HIF‐1α. Notably, the HIF‐1α protein band is only present in the immunoprecipitates of the control cells. (**B**) Representative Western immunoblot of lysates of SAS cells harbouring the TMEM207 expression vector or control cells. The TMEM207‐expressing cells (labelled ‘TMEM207′) exhibited detectable levels of HIF‐1α, even under normoxic conditions, whereas a weak or no HIF‐1ɑ band was detected in control SAS cells (labelled ‘Mock’) under normoxic conditions. These experiments were repeated using different clones, and consistent results were obtained.

We performed a co‐immunoprecipitation assay to determine whether TMEM207 disrupts the binding of WWOX to HIF‐1ɑ using SAS (a human tongue squamous carcinoma line) cells transfected with a TMEM207 expression vector. SAS cells transfected with empty vector were used as controls. The TMEM207‐expressing cells displayed decreased binding of WWOX to HIF‐1α (Fig. 3A) compared to controls. Interestingly, SAS cells harbouring the TMEM207 expression vector exhibited significant levels of HIF‐1α in Western immunoblotting, even under normoxic conditions (Fig. 3B). By contrast, a weak or no HIF‐1ɑ band was detected in control SAS cells under normoxic conditions (Fig. 3B).

These data suggest that the binding of TMEM207 to WWOX inhibits the interaction between WWOX and HIF‐1α, thereby hampering the degradation of HIF‐1α under normoxic conditions.

### Enforced expression of TMEM207 increased tumour progression in a xenoplant assay

**Figure 4:**
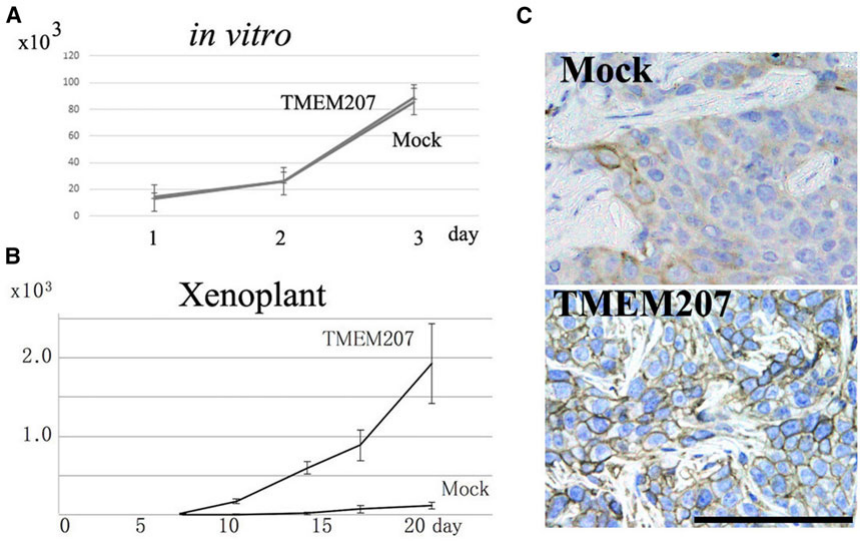
TMEM207 expression increases SAS tongue squamous carcinoma cell growth *in vivo* and GLUT‐1 protein expression. (**A**) Enforced TMEM207 expression did not affect cell growth *in vitro*. SAS cells were cultured on tissue culture dishes in triplicate. Data are expressed as means ± S.D. (*n* = 3). Measurements of live cells were taken at 24, 48 and 72 hrs. No significant differences in cell growth were found between TMEM207‐expressing and control SAS cells. The experiment was repeated two times using different clones, and consistent results were obtained. (**B**) Enforced expression of TMEM207 increased the growth of SAS cells in a xenoplant assay. Representative results obtained for transfected clones are shown. Similar results were obtained using different transfected clones. Values are presented as means ± S.D. (*n* = 3). (**C**) GLUT‐1 immunoreactivity in the xenoplant assay was significant in TMEM207‐expressing SAS cells but sparse in control cells.

We also used the TMEM207‐expressing transfected SAS cells to assess whether enforced TMEM207 expression may facilitate the growth of OSCC. Even though no significant differences in *in vitro* proliferation were observed between TMEM207‐expressing and control SAS cells (Fig. 4A), xenoplant assays revealed that the former TMEM207‐expressing SAS cells formed significantly larger tumours compared to the latter (Fig. 4B). We obtained similar results using another independent TMEM207‐expressing SAS cell clone.

Notably, GLUT‐1 expression was ubiquitous in xenotransplanted TMEM207‐expressing SAS cells (indicated as ‘TMEM207′ in Fig. 4C). By contrast, GLUT‐1 expression was sparse in the central region of xenotransplanted control SAS cells (indicated as ‘Mock’ in Fig. 4C).

### siRNA‐mediated silencing of *TMEM207* impaired *GLUT‐1* expression in CHU‐2 and SCC‐9 cells

**Figure 5:**
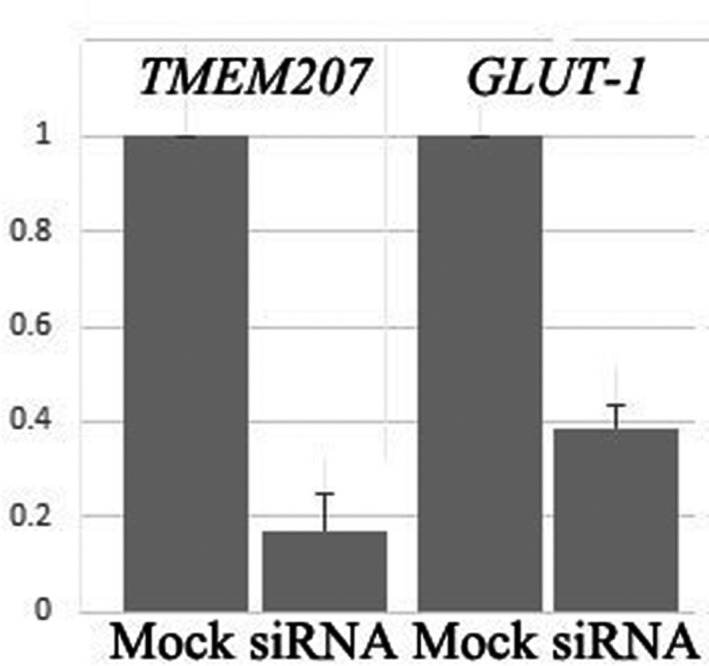
Down‐regulation of TMEM207 expression decreases *GLUT‐1* mRNA in cultured OSCCs. Representative quantitative RT‐PCR data are shown. Down‐regulation of TMEM207 significantly decreased *GLUT‐1* mRNA in both CHU‐2 and SCC‐9 cells. Cells were transfected with SI04341981 (Qiagen, indicated as ‘si‐*TMEM207’*) or control GFP‐siRNA (indicated as ‘si‐Mock’). Results using SCC‐9 cells are presented as means ± S.D. (*n* = 3). Similar results were obtained using CHU‐2 cells and a different siRNA (SI04277770).

Next, we examined whether the down‐regulation of TMEM207 in OSCC alters the status of GLUT‐1, which is a representative HIF‐1ɑ‐targeting molecule, by knocking down *TMEM207* expression in two OSCC cell lines, CHU‐2 and SCC‐9. *TMEM207* silencing with either of the two siRNAs significantly down‐regulated *GLUT‐1* mRNA expression in both cell lines. Results for SI04341981 and SCC‐9 cells are shown in Figure 5.

## Discussion

In the physiological state, TMEM207 expression is relatively restricted to intestinal goblet cells and renal tubular cells 26. However, various cancers aberrantly express TMEM207. Our immunohistochemical staining results confirmed TMEM207 expression in many OSCC cases. Notably, in the TMEM207‐positive OSCC cases, not only the invasive squamous cell carcinoma cells but also the neighbouring dysplastic mucosal cells exhibited TMEM207 immunoreactivity. However, the localization of the signal differed; TMEM207 in the invasive cells was typically cytoplasmic, whereas it was mainly found at the membranes of the dysplastic mucosal cells. At this point, it is worth mentioning that although TMEM207 was initially identified as a novel secretory and transmembrane protein by the Secreted Protein Discovery Initiative 26, a subsequent bioinformatic analysis suggested that TMEM207 is localized to the endoplasmic reticulum. In fact, many gastric signet‐ring cell carcinoma cells exhibit cytoplasmic expression of TMEM207 14.

As demonstrated in Figure 1A, TMEM207 immunoreactivity was found in the membrane of dysplastic epithelial cells neighbouring invasive squamous cell carcinoma cells, while no significant TMEM207 immunoreactivity was detected in non‐tumorous oral mucosal epithelium (Fig. 1D). Recent advances suggest that TMEM207 may participate in proper processing of an adipokine protein, Intelectin‐1, in colon mucosa at the surface membrane 16, 27. We speculate that an Intelectin‐1‐mediated signalling cascade, which has been recently highlighted to be involved in carcinogenesis 27, aberrantly occurs in the surface membrane of pre‐cancerous epithelial cells. However, future studies are needed to unravel the pathobiological properties of TMEM207 in the surface membrane of pre‐cancerous oral mucosal epithelial cells.

A proximal ligation assay demonstrated the colocalization of WWOX and TMEM207 in invasive squamous cell carcinoma cells. We speculate that the aberrant expression of TMEM207 might be an early event during oral carcinogenesis, wherein cytoplasmic TMEM207 binds to WWOX to attenuate the tumour suppressor function of the latter in invasive squamous cells.

TMEM207 has high protein homology to the vesicular overexpressed in cancer pro‐survival protein 1 (VOPP1) 28, 29, which acts as an oncogenic factor in tobacco‐related human squamous cell carcinoma; the encoding gene is located on human chromosome 7p11.2, which is often amplified in human squamous cell carcinoma 28. On the other hand, *TMEM207* is located on human chromosome 3q28, near *TP63*. Because the 3q26.3‐qter region is also often amplified in OSCC 30, the aberrant expression of TMEM207 in OSCC may be associated with gene amplification, similar to the case of *VOPP1*; however, additional studies are needed to determine the exact molecular mechanism of TMEM207 expression.

WWOX is believed to exert its tumour suppressor role *via* binding to various molecules. Although *WWOX* is located at the common chromosomal fragile site FRA16D 31, impairment of both alleles is rare 32. A previous study demonstrated that even though a loss of WWOX is found locally in OSCC, many invasive OSCC cells still express WWOX at detectable levels as shown by immunohistochemical staining 13. Based on our immunohistochemical staining results, WWOX was expressed in 22 of 30 OSCC tissue specimens. We speculate that TMEM207 might play a role in the loss of WWOX function in a substantial number of invasive OSCC cells that still express WWOX. The present co‐immunoprecipitation assay demonstrated that enforced TMEM207 expression resulted in a decrease in the WWOX‐HIF‐1α complex binding in cultured OSCC cells. Even in normoxic conditions, HIF‐1α expression was detected in cells with enforced TMEM207 expression. Moreover, the siRNA‐mediated silencing of *TMEM207* decreased *GLUT‐1* mRNA, which is a representative HIF‐1ɑ‐targeting molecule, in cultured OSCC cells. Combined, these results suggest that TMEM207 expression contributes to carcinogenesis in many OSCC cases by inhibiting the WWOX‐mediated regulation of the HIF‐1α pathway.

In summary, the results of the present study revealed new pathobiological properties of TMEM207 in OSCC. TMEM207 was aberrantly expressed in many OSCCs, and this aberrant expression might contribute to aerobic glycosylation and facilitate OSCC growth, possibly by abrogating WWOX function. Thus, TMEM207 is a candidate target molecule for the treatment of OSCC.

## Conflict of interest

All contributing authors declare no conflict of interests.
